# A Short-Term Evaluation of the Eat and Exercise to Win Program for Adults with Intellectual and Developmental Disabilities

**DOI:** 10.3390/nu16183124

**Published:** 2024-09-16

**Authors:** Laura Nabors, Anne Bauer, Kara Ayers, Brandon Workman, Melinda Butsch Kovacic, Seung-Yeon Lee

**Affiliations:** 1 College of Education, Criminal Justice and Human Services, University of Cincinnati, Cincinnati, OH 45221, USA; baueram@ucmail.uc.edu (A.B.); workmabn@ucmail.uc.edu (B.W.); 2 Cincinnati Children’s Hospital Medical Center, Cincinnati, OH 45229, USA; kara.ayers@cchmc.org; 3 College of Allied Health Sciences, University of Cincinnati, Cincinnati, OH 45267, USA; butschms@ucmail.uc.edu (M.B.K.); lee2so@ucmail.uc.edu (S.-Y.L.)

**Keywords:** healthy eating, exercise, health education, eat and exercise to win program

## Abstract

(1) Study Aim: Adults with intellectual and developmental disabilities (IDD) face a multitude of chronic health risks related to obesity, including diabetes and heart disease. Day adult service programs offer unique opportunities for improving and monitoring the health of this vulnerable group. To promote exercise and healthy eating habits among adults with IDD, the Eat and Exercise to Win Program (EE-2-Win) was offered weekly at two locations over 8–9 months. (2) Methods: Using daily logs, staff assessed outcomes of 26 program participants, including changes in knowledge, eating and exercise habits, and water consumption. Participants’ weights were measured, and their lunches were photographed at baseline and 3 months. (3) Results: While participants’ weights did not significantly differ at 3 months, assessing photographs indicated that participants’ lunches included more fruits and vegetables. Staff surveys indicated that participants had greater knowledge of MyPlate and were consuming more water at three months. Survey responses indicated that staff perceived the program to be valuable overall, and challenges in learning and assessment were reported given differences in the cognitive abilities of adults with IDD. Staff also suggested engaging home caregivers in the program, as they often make dietary decisions. (4) Conclusions: Overall, results confirmed that the EE-2-Win Program positively impacted healthy eating and exercise. Future work, however, with both staff and caregivers is needed to further optimize the program.

## 1. Introduction

In 2019, 7.39 million people in the United States were estimated to have intellectual or developmental disabilities [1]. Adults with IDD are more likely to be overweight or obese than adults in the general population [2] due to lack of physical activity and poor nutrition [3]. They tend to have a low intake of fruits and vegetables [4,5,6] and high sugar-sweetened beverage or soda consumption [7]. In addition, adults with IDD are about 50% less likely to engage in physical activity compared to adults in the general population [8]. As a result, they encounter increased risks for diabetes, heart disease, and other chronic health conditions [9,10]. There is a need for health education programming developed for adults with IDD, such as interventions for obesity, to promote healthy eating and exercise in community programs, such as adult day services or programs [11,12,13,14].

Individuals with IDD often have substantial limitations in self-care [3] as well as significant cognitive delays and use adult day services. Adult day services (ADS) are defined as “a system of professionally delivered, integrated, home- and community-based, therapeutic, social and health-related services provided to individuals to sustain living within the community” [15]. ADS programs are based in the community and provide groups of adults who need supervision care during the day. ADS may include transportation to and meals at the program as well as providing activities to facilitate socialization, exercise, or new knowledge provision for adults. ADS may also offer medical care and therapies, such as speech or occupational therapy [16]. Thus, adult day services provide opportunities to track and improve the health of adults with IDD [17].

There is an urgent need for health education programming developed for adults with IDD, such as interventions for obesity, to promote healthy eating and exercise, especially in community settings [11,12,13,14]. Participating in health education programs has been correlated with improvements in healthy cooking habits [12], increased fruit and/or vegetable intake [18,19] and more frequent involvement in physical activity [18]. Salomon et al. (2023) [20] recommended developing practical educational messages and activities to involve adults in their own behavior changes (e.g., food choices), and this notion is incorporated in the EE-2-Win Program [18]. However, some intervention studies have not found significant changes or found only minor changes related to health education [21] or have only assessed long-term changes (at intervals of six months or more [22]), and a further evaluation of shorter-term impact of health education programs on behavior and knowledge change in community settings is needed. Research on the short-term impact of the EE-2-Win Program on healthy eating and exercise is lacking, and this pilot study provides initial information about the short-term impact of this program.

The EE-2-Win Program is grounded in adults selecting their own goals and directing their own behavior change. This is synonymous with self-determination theory, which suggests that empowering individuals with IDD to identify their goals (in our case, healthy eating and exercise goals) may be most effective in fostering independence and change [23,24,25]. Improving individual agency in changing attitudes and behaviors is also consistent with Social Cognitive Theory [26,27]. The program utilizes a motivational interviewing approach, which supports self-efficacy for change, and is a non-judgmental method for developing goals in an incremental approach to adapt behaviors [28]. This method can be especially helpful with those who are ambivalent about making changes [29] or, like many adults with autism and intellectual disabilities, have difficulty overall with change and as a result, resist. Thus, a focus on empowering individual choices and prioritizing small changes helps adults with IDD become their own advocates for changes in their eating and exercise behaviors [22,30]. This approach has resulted in positive changes in other intervention programs for adults with IDD [31].

Assessing the impact of interventions can be a challenge, given the differences in abilities among adults with IDD. However, image-assisted dietary-assessment methods have evolved with the common usages of smartphones and other available digital technologies to overcome the limitations of self-report data in traditional dietary-assessment methods. Digital photography, which can be used to assess food provision, food selection, plate waste, and food intake [3,32,33,34], as well as documenting change in eating behaviors when before and after photographs are used, is a promising assessment tool [12,35]. Complex food records, specifically photographing meals to assess changes in eating behaviors, are also promising [12,35]. However, adults with IDD may have difficulty recording photographs. Other assessment methods, such as counting foods in lunches, may improve opportunities for staff members to learn to record data. Bergström et al. (2013) [4] had adults with IDD take photographs of meals on two weekdays and one weekend day before and after implementing a general health program (Driver’s License for Health). However, they found significant missing data and that some adults with IDD did not wish to complete the assessments. Salomon et al. (2023) [20] also observed similar missing data during the evaluation of a pilot study of the “Get Healthy!” eating and exercise program. Only two out of six participants with IDD completed the assessment by taking photographs. Thus, a simpler assessment method is needed, and evaluators or staff members may need to record photographs of lunches. Changes in lunchtime meals for adults participating in the EE-2-Win Program have not been measured and use of photographs of lunches allowed for a pilot assessment.

The current study aimed to evaluate the impact of participating in the program for 3 months on the changes in eating fruits and vegetables in lunches, drinking more water, and engagement in light exercise in ADS programs for adults with IDD. Change in weight was assessed, although change at a three-month interval was not expected. In addition, staff members’ perceptions of the program were examined.

## 2. Study Design

A single-arm study was employed to evaluate the impact of the EE-2-Win Program on eating, beverage consumption, and exercise changes for adults with IDD. The Institutional Review Board at the University of Cincinnati approved this study (#2020-0424).

### 2.1. Participants and Recruitment

Twenty-six adults with IDD and six staff members in two ADS programs consented to participate. Adults with IDD had intellectual and developmental disabilities and cognitive delays requiring daily supervision. Disability status was determined by family reports for all participants. All had cognitive delays that are considered severe [36] and required daily assistance with self-care activities and safety. Eleven adults with IDD were recruited at Site 1 and fifteen were recruited at Site 2. Six staff members were also recruited in the two programs.

L. N. recruited participants. Participants with IDD signed the picture-based assent form after learning about this study. A staff member at each site observed the assent process and witnessed assent forms. If participants had a guardian, the guardian also reviewed and signed the consent form. Staff members also signed forms, documenting their consent to participate.

### 2.2. Intervention: EE-2-Win

The EE-2-Win centers adults with IDD in health education while focusing on improving healthy eating and exercise through use of motivational interviewing with an emphasis on adults setting their own health change goals [18,22,30]. Adults with IDD learned about roadblocks (something that gets in the way) of healthy eating and exercise and the group leaders shared their personal roadblocks. Participants also discussed ways to overcome health roadblocks. Adults with IDD reviewed picture-based slides with coaches (L. N. and an undergraduate and graduate student who were research assistants) in weekly lessons and set change goals at the end of the lessons. Staff observed lessons and discussed information during and after lessons with adults with IDD.

Lessons (delivered using picture-based slides) focus on describing and applying MyPlate (see https://www.myplate.gov/ accessed on 14 September 2024), a guide from the U.S. Department of Agriculture for determining meal composition, and reviewing MyPlate breakfasts, lunches, dinners, and snacks. The five food groups are reviewed and recipes for smoothies, healthy meals, and snacks are presented. The vitamins in foods, the importance of eating the rainbow of fruits and vegetables, and reasons for healthy eating (improving weight control and reducing risks for diabetes and heart problems) are among the topics discussed. Participants played a “This or That” Game developed by L. N. and students to select the healthiest food option from choices of two different options. Portion sizes for the five food groups are reviewed and examples of large and “right-sized” adult portions are discussed with participants. Understanding the role of fast food, processed foods, and highly processed snack foods is reviewed (see Nabors et al. (2021) [18] for a review). Drinking less soda or replacing soda with sugar with diet soda or water with fruit is emphasized. Participants also learn about the value of exercise and stretching and how this can improve mood, sleep, and strength of muscles and bones. They review the importance of exercising 150 min per week (30 min per day and five days a week). Participants learn and practice different stretches with the coaches. They participate in chair exercises (using videos downloaded from the internet) or walk with coaches. Lessons are delivered for one hour each week.

Staff are provided with the manual and lessons for the program. For this study, staff encouraged adult participants to eat healthily and exercise during the week, reminding adult participants about healthy eating tips and exercising with them in walking clubs and other group exercises.

### 2.3. Implementation of the Intervention

Adults with IDD and staff members in the program attended weekly 60 min sessions from the EE-2-Win Program [18] led by L. N. and a different student at each site. Sessions were delivered before lunch at each site. Adults consumed lunches that were packed at home at each site. L. N. and students delivered lessons using PowerPoint slides from the manual for the EE-2-Win Program, focusing on MyPlate; eating more fruits and vegetables; healthy meal and snack choices; the description of high-carbohydrate, non-nutritional foods (i.e., junk food); eating a variety (the “Rainbow”) of different colored fruits and vegetables; and understanding appropriate portion sizes. The first 30–35 min of lessons focused on healthy eating and the last 25–30 min focused on stretching, walking, or participating in chair exercises (e.g., Chair One Fitness; https://chaironefitness.com/ accessed on 14 September 2024). In five of the lessons, information on roadblocks to healthy eating and ideas for overcoming them were discussed. Staff members attended the 12 weekly lessons. Staff at the sites rated fidelity to implementing material in the manual at 90–100% across lessons. Three newsletters, reviewing program activities, were sent to guardians every three to four weeks. L. N. trained staff members at each site on recording fruits and vegetables in lunches and whether adults with IDD were drinking water and engaging in the exercise program during four days of the week (data were not recorded on the day lessons were delivered in the programs).

### 2.4. Data Collection

Adults with IDD were weighed before the intervention began and at three months using a Tanita WB-3000 Digital Physician’s Scale (660 lb. capacity). The Tanita scale provided data on weight in lbs. and Body Mass Index. Three of the adults with IDD could not remain on the Tanita scale, one was on a walker (he appeared to be overweight), and two participants who appeared to be overweight refused to step on the scale. Adults with IDD often had difficulty standing straight and thus height was not recorded using the stadiometer on the Tanita scale. Body weight was classified using criteria from the Centers for Disease Control and Prevention [37]—BMI of less than 25 (24.9 and below) is average weight and BMI of 25–29.9 is overweight, and 30 and greater is obese. Pictures of adults’ lunches were taken, using cellular telephones, at lunchtime at baseline and after 3 months.

Staff members responded to surveys developed for this study (to assess behavior and knowledge change in adults with IDD and staff perceptions of the program) at the end of the third month of the program. They estimated how much each participant ate at lunch (0–100%). Staff members answered two short-answer questions—(1) they reported if they had made any changes because of observing the program, and (2) discussed whether they reinforced the program during the week. They answered two questions on 4-point scales (none (1), a little (2), some (3), a lot (4)), where they rated knowledge and behavior change in participants. Staff members recorded behavior changes they observed related to the EE-2-Win Program. Finally, they responded to two questions assessing the need for the program and whether they would use the program in the future on 6-point Likert scales from strongly disagree (1), disagree (2), slightly disagree (3), slightly agree (4), agree (5), to strongly agree (6).

Staff members also recorded engagement in exercise while in the program and whether adults with IDD had fruit or vegetables in their lunches and drank water during the day (while in the program) for four of the five weekdays.

### 2.5. Data Analyses

Descriptive statistics presented information on adults with IDD. Friedman’s tests were used to compare change in Mean Ranks from baseline to three months for weight and weekly records of fruit, vegetables, water, and exercise recorded by staff members at Site 2 [38,39]. Analyses were performed using IBM SPSS Statistics (Version 29.0) and a significance level of 0.05 was statistically significant. However, a Bonferroni correction was applied for multiple tests for staff reports of fruit, vegetables, water consumption, and exercise involvement. Descriptive statistics were used to summarize the frequencies of fruit, vegetables, water, soda, salty snacks, and sweet treats in photographs of lunches. The percentage agreement between coders was calculated for records of frequencies of fruit, vegetables, water, soda, salty snacks, and sweet treats in photographs of lunches by L. N. and an undergraduate student. Kappa was used to assess agreement on the number of high-carbohydrate foods in lunches coded by L. N. and an undergraduate student. A Friedman’s test was used to examine coder agreement for the number of high-carbohydrate foods in the lunches. Descriptive statistics were used to present staff members’ reports of perceptions of change for themselves and adults with IDD.

### 2.6. Coding Lunch Pictures

L. N. and an undergraduate student worked together over two meetings to code the presence of fruit, vegetables, water, and soda; portion size of salty snacks; portion size of foods high in sugar (treats); and number of high-carbohydrate foods in pictures of lunches. L. N. and an undergraduate coder counted the number of high-carbohydrate foods in photographs of lunches. High-carbohydrate foods were defined as breads, snacks, and treats that were high in carbohydrates. L. N. coded 100% of the lunch photographs, and an undergraduate coder reviewed 50% of the lunch photographs. An open coding process to search for key themes about health and areas for improvement of lunches was used to review data in photographs. L. N., A. B., and an undergraduate student reviewed lunch photographs and discussed their impressions and came to a consensus about impressions for improving the “health” value of foods packed in lunches.

## 3. Results

### 3.1. Demographic Information

Twenty-six adults with IDD (fourteen males and twelve females) were recruited at the two sites. Their ages ranged from 24 to 64, with an average age of 36.46 years (*SD* = 11.40). Three were African American and 23 were white. Six (five females, one male) staff members working with the adults in the ADS programs also participated. One staff member was Hispanic, one was African American, and four were white. Age was not recorded for staff members. Demographic information for adults with IDD for the sites is presented in Table 1.

### 3.2. Findings for Food and Beverage Intake in Photographs of Lunches

Percent agreements between coders and percentage of fruits, vegetables, water, soda, salty snacks, and sweet foods high in sugar as well as the percentage of times each category was present in photographs of lunches are presented in Table 2.

Site 1. Three (42.86%) of the seven adults with IDD had healthy changes in their lunches by three months (Table 1; one adult did not have a three-month assessment). One male and one female had more fruit in their lunches and one male had water rather than soda. And another male did not have a soda in his lunch at three months.

Site 2. Six of the adults with IDD (50%) had healthy changes in their lunches by the three-month assessment, while six did not (Table 1; two adults did not have a 3-month assessment). Three of the six were packing more fruit in their lunches (one male, two females). Two females brought fewer cookies in their lunch (from four to three cookies). Two of the adults with IDD, a male and a female, packed veggies more often (carrots and celery). One male was drinking more water, and one female did not have a salty snack. Five fewer individuals had sweet treats (foods high in sugar) at the three-month assessment. Importantly, some also decreased the number of sweet foods that were high in sugar that they packed. Specifically, the number of sweet foods high in sugar decreased from two to one for three females and from four to one for one male.

Results of the Friedman’s test were not significant (*p* = 0.06). Agreement was assessed using Cohen’s Kappa. The Kappa was 0.673 for the number of high-carbohydrate foods at baseline and 0.615 at three months. The inspection of mean scores indicated a slight reduction in the number of high-carbohydrate foods from baseline (*M* = 2.65, *SD* = 1.41, range = 1–5 high-carb foods; Mean Rank = 1.68) to three months (*M* = 2.12, *SD* = 1.17, range = 0–4 high-carb foods; Mean Rank = 1.32).

Regarding qualitative impressions, the three coders were concerned with portions, thinking portion sizes for some of the adults with IDD were exceptionally large. Also, most (about 85%) typically had both a salty snack and at least one food high in sugar. Two of the males with IDD who were overweight had two sandwiches in their lunches. Coders also noted that salty snacks packed in ‘baggies or plastic bags’ were larger portions than snack-size bags of potato chips. For some adults with IDD who were overweight and obese, the snack and treat portions remained high, especially if portions of sweet foods high in sugar were packed in a sandwich bag rather than in a pre-packaged, small snack bag.

Coders had several suggestions to improve “health” related to packing healthier lunches (eliminating soda and sugary drinks, replacing salty snacks with healthier snacks, and replacing sandwich bread; wheat bread instead of white bread). Coders recommended eliminating soda and drinks with sugar (e.g., “Capri Sun”) from lunches. Coders suggested replacing salty snacks (e.g., chips and Cheetos) with “Veggie Straws”, air-popped popcorn, baked snacks (e.g., ‘Baked Lays’), puffed popcorn, carrots, or celery. They also recommended replacing white bread on sandwiches with wheat bread and packing turkey and ham rather than processed lunch meat like bologna. Portion sizes of foods high in sugar should be reduced and fruits introduced as treats or dessert in lunches.

### 3.3. Weight Status

There were differences between individuals and sites for weight (see Table 1). Results indicated that mean weight did not significantly change from baseline to three months at both sites. There were missing data at both sites for weight assessment at three months.

### 3.4. Staff Report

Site 1. Staff members recorded a change in the number of times exercising (walked or stretched) per week and a change in the number of glasses of water drunk while in the program throughout the day. Staff members’ records did not indicate changes in exercising or drinking water. Data were incomplete.

In terms of social validity, however, staff members reported that keeping the logs helped them learn of two adults with IDD who needed to “move” and “exercise more” and they specifically identified five adults with IDD who needed to drink more water. Staff members reported that they would keep recording drinking water as this was helpful to their knowledge.

Site 2. Staff members at this program kept daily records (four days per week; no data were recorded the day of the week that the lesson was delivered, which was Tuesday) for twelve weeks. Week one was the baseline and during the next 11 weeks, the intervention was in progress.

Results of the Friedman’s tests (see Table 3) indicated significant change over time for intake of fruits, vegetables, and water, and days exercising.

Results indicated improvements in fruit and vegetable intake, when considering a Bonferroni correction of 0.0125 for the four tests. However, an inspection of Table 3 indicates that gains in water consumption and exercising were also evident. Regarding social validity, staff members continued to record fruit, vegetables, water, and exercise after data recording was completed.

### 3.5. Staff Members’ Perceptions of the Eat and Exercise to Win Program

Site 1. Two female staff members provided data about their perceptions. They reported that one adult ate 80–85% of his lunch and the others ate 100% of their lunches. Concerning personal changes, one female staff member reported eating less fast food and stretching and exercising more and both females reported eating smaller portions because they attended the EE-2-Win Program. Both women reported that they reinforced learning from the EE-2-Win groups weekly through encouraging exercise and eating more fruit. Both staff members reported that they observed changes in knowledge of adults with IDD; one reported some (rating of 3 out of 4) knowledge change and the other reported a little (2) knowledge change. Both females reported behavior change with one reporting a little positive change (2) and the other reporting some (3) positive change. They both reported that the adults with IDD were exercising more. However, both females questioned whether adults with more significant cognitive delays could follow some of the information provided. In terms of the need for the program, the average rating was 5 (4 and 6, respectively), indicating agreement that a program was needed, and both females agreed that they would use the intervention in the future, with an average rating of 5.5 (5 and 6, respectively).

Site 2. One male and three females rated the program. They reported that one adult ate 80% of his lunch and another ate 90%. Six adults ate 100% of their lunches. Regarding personal changes, staff members reported improving what they packed for lunch and exercising more. One of the staff members was losing weight. All staff discussed and referenced material from the lessons in small group meetings during the week. One staff member, a female, stated that she was discussing eating vegetables more because they had planted a garden. Another staff member reported that they had fruit at their Valentine’s Day party for the first time and she reported that adults with IDD proudly showed staff their “healthy” lunches on a regular basis. All four staff members reported changes in adults’ knowledge and behavior change, with three staff members reporting some (3) behavior and knowledge change and one reporting a little (2) behavior and knowledge change. In terms of the need for the program, all the staff members agreed that there was a need for the program (average rating of 5) and all four agreed (average rating of 5) that they would use the program in the future.

## 4. Discussion

Results indicate that the EE-2-Win Program had a positive impact on healthy eating and exercise after three months, extending the literature by showing that the intervention had positive short-term impact. This is important as research indicating whether healthy eating and exercising programming has a positive impact at intervals shorter than six months is needed. At each site, there were more fruits and vegetables in lunches at three months and adults with IDD were exercising more and drinking more water at one of the sites. These results were consistent with previous results after a year in the EE-2-Win Program [18,22,30] and other research showing that improving knowledge about healthy eating and engaging in exercise promote the health of adults with IDD, but over longer intervals [2,20,35]. Staff members reported a need for the program, and most of the adults with IDD had BMIs in the overweight or obese range, which was consistent with previous research indicating that being overweight is a problem and there is a need for interventions for adults with IDD [2,13,14]. However, there were between-site differences as well, and at one site, staff members reported that those with severe delays had difficulty understanding some lessons, suggesting that further research with the EE-2-Win Program, with adults of differing cognitive abilities, is needed. Also, results suggested that staff members were participating in the program, making changes along with the adults in their program, and reinforcing the program during the week. This was positive, and more information on how programming influences staff and how they reinforce and continue to present information from the EE-2-Win Program is needed.

Improving eating habits at lunch for adults with IDD and, if needed, staff members could be the beginning of healthy changes related to eating more fruits and vegetables. Further, improving fruit and vegetable intake and involvement in light physical activity has the potential to become a healthy lifestyle change that leads to weight loss [10,40]. Change is vital as an overweight status is related to health concerns, such as diabetes and cardiovascular disease, for adults with IDD [10,40]. Also, results indicated that staff positively changed their eating habits, and improving staff knowledge and behaviors can be an impetus for them to teach and help adults with IDD to change. There was some positive change in the current study after three months (i.e., 12 lessons), although there was no weight loss at our three-month assessment. To this effect, many programs have assessed change at year-long [12] or six-month intervals [2,35], and we suggest that change in healthy food intake and weight loss should be assessed at short-term and longer-term intervals in future studies.

## 5. Limitations

Several factors limited the generalizability of study findings. For instance, the sample size was small, but it was encouraging that there was positive change. Moreover, the samples were recruited at two sites, and there were differences in the groups between sites (see Table 1), but the common thread was that all the adults had IDD. Site differences could be viewed as enhancing the generalizability of findings, as there were some improvements in healthy eating at both sites. There was not a control group, but the sites wanted all adults to have an opportunity to participate. Adults with IDD ranged from 24 to 64 years of age and there could have been developmental differences in their responses and the changes they made, based on sociocultural influences. Also, we did not assess adults’ cognitive functioning, and adults with more significant delays may not have been able to comprehend some of the programming; however, staff did indicate knowledge change for adults with IDD, which is promising. Details on the impact of other disabilities are lacking, as is information on whether adults were making decisions independently, and this information is needed in future studies. We did not have data on who packed the lunches, although anecdotal data suggested that adults with IDD were involved. Learning who packs what foods and educating all involved in packing lunches will be important. Obtaining information about lunch and exercise in the ADS program is only part of the picture and obtaining information about eating and exercise at home will improve knowledge and point to additional areas for intervention. A few of the adults did not get their weight assessed and did not allow pictures of their lunches to be recorded. In future research, uncovering reasons for this and finding a way to assess the progress of people who do not have weight data and do not want photographs recorded will be important.

## 6. Conclusions

The EE-2-Win Program had short-term benefits, indicating that participating in the program resulted in increased fruit consumption and was useful for adults with IDD. These results were consistent with previous research assessing change over longer intervals [18,22,30]. According to staff members, adults with IDD expressed a sense of pride when they packed healthy lunches, and staff members from both sites felt that they would use the material from the program in the future. Thus, health programming for this pilot study was successful in improving self-efficacy for healthy eating and exercise and involving staff and adults with IDD working as a team for change, which has the potential for long-term health changes. Involving caregivers in the program may improve opportunities for positive change as they more often make dietary decisions. Using photographs and counting the change in the number of healthy foods in lunches was an assessment method that staff can use to track change, and thus this study highlights a practical assessment method, which is critical to moving assessment into community settings. Recipes were sent home and perhaps cooking the recipes in programs on a family cooking night would be a way to reach caregivers and provide them with program information. Reaching parents via Zoom groups or personalized text messages, emails, or telephone calls may be other methods for providing them with more personalized information than using newsletters (as was the case for this study). Obtaining information from caregivers would strengthen study findings to determine if the adults were educating the parents/guardians or if the latter were changing meals at home or exercising more due to education from newsletters or some other reason. It remains encouraging, however, that there was change without direct involvement of parents or guardians. Given that this was a pilot study, future research with a control group is needed to verify that change resulted from program participation. Determining whether participating in the program is related to long-term change in meals and exercise at home also remains important, and assessing progress of a control and intervention group over time will be important to understanding program impact.

## Figures and Tables

**Table 1:** Demographic Data for Adults with IDD for Site 1 and Site 2.

Group	Variable	Variable Category	Site 1 *n*	Site 2 *n*
Adults with IDD	Sex			
		Males	8	6
		Females	3	9
	Mean Age		*M* = 33.09 (*SD* = 8.4), range of 24–55	*M* = 38.93 (*SD* = 12.88)
	Race			
		African American	3	0
		White	8	15
	Primary Diagnoses			
		ASD	3	2
		Down Syndrome	2	5
		Developmental Delay	2	5
		Periventricular Leukomalacia	1	
		Microcephalus	1	
		Cerebral Palsy	1	1
		Traumatic Brain Injury	1	2
	Mean Weight		*M* = 215.80 (*SD* = 72.91), range of 130–363.40 lbs.	*M* = 204.76 (*SD* = 59.72), range of 121.20–303.80
	BMI		*M* = 35.56 (*SD* = 11.00), range of 21.80–57.1	*M* = 32.15 (*SD* = 9.38), range of 19.00–47.70

Notes. At Site 1, there was 1 male with IDD who used a walker and at Site 2, there were 2 females with IDD using walkers.

**Table 2:** Percent of Intake of Selected Food Groups in Photographs of Lunches at Sites 1 and 2 at Intake and Three Months.

Variable	Percent Agreement	Site 1 (*n* = 8)		Site 2 (*n* = 14)	
		Baseline % (*n*)	3 Months % (*n*)	Baseline % (*n*)	3 Months % (*n*)
Fruits	95.9%	37.5% (3)	71.4% (5)	28.6% (4)	58.3% (7)
Vegetables	90.3%	25.0% (2)	28.6% (2)	21.4% (3)	41.6% (5)
Water	93.0%	50.0% (4)	71.4% (5)	7.1% (1)	13.3% (2)
Soda	100.0%	37.5% (3)	14.3% (1)	42.9% (6)	54.5% (6)
Salty Snacks	90.3%	87.5% (7)	89.7% (6)	85.7% (12)	91.7% (11)
Sweet Foods High in Sugar	89.0%	50.0% (4)	71.4% (5)	78.6% (11)	50.0% (6)

Notes. Only three of the adults with IDD did not eat 100% of their lunches, eating about 80–90% of their lunches per staff report. Three of the males with IDD at Site 1 did not have photographs of lunches. Two had left the day program and one had broken his knee cap. One female with IDD at Site 2 refused to have pictures of her lunch recorded.

**Table 3:** Results of Changes in Fruit, Vegetable, Water, and Exercising Over Four Weekdays from Baseline to Three-Month Assessment at Site 2.

Variable	Friedman’s Test Results Chi-Square Value, *p* Value	Baseline *M*(*SD*), Range, Mean Rank (MR)	Three Months *M*(*SD*), Range, Mean Rank (MR)
Fruits	28.99, *p* = 0.002	*M* = 2.25 (*SD* = 0.96), range = 1–3, MR = 1.13	*M* = 3.75 (*SD* = 0.5), range = 3–4, MR = 6.35
Vegetables	27.83, *p* = 0.003	*M* = 2.0 (*SD* = 0.83), range = 1–3, MR = 1.5	*M* = 3.5 (*SD* = 0.5), range = 3–4, MR = 6.5
Water	20.97, *p* = 0.034	*M* = 2.25 (*SD* = 0.5), range = 2–3, MR = 1.63	*M* = 3.25 (*SD* = 0.96), range = 2–4, MR = 5.13
Exercising	22.56, *p* = 0.02	*M* = 2.75 (*SD* = 0.5), range = 2–3, MR = 1.75	*M* = 3.75 (*SD* = 0.5), range = 3–4, MR = 6.38

Notes. Bonferroni correction of 0.0125. Assessment on 4 out of 5 weekdays. Degrees of freedom for Chi-square tests were (1, 11) for all four analyses.

## Data Availability

Data are available on request from Laura Nabors at naborsla@ucmail.uc.edu. Data are part of an ongoing study and are sensitive. Data will be preserved for five years after publication.
